# Fidgety Philip and the Suggested Clinical Immobilization Test: Annotation data for developing a machine learning algorithm

**DOI:** 10.1016/j.dib.2021.106770

**Published:** 2021-01-17

**Authors:** Melvin Chan, Emmanuel K. Tse, Seraph Bao, Mai Berger, Nadia Beyzaei, Mackenzie Campbell, Heinrich Garn, Hebah Hussaina, Gerhard Kloesch, Bernhard Kohn, Boris Kuzeljevic, Yi Jui Lee, Khaola Safia Maher, Natasha Carson, Jecika Jeyaratnam, Scout McWilliams, Karen Spruyt, Hendrik F. Machiel Van der Loos, Calvin Kuo, Osman Ipsiroglu

**Affiliations:** a H-Behaviours Research Lab, BC Children's Hospital Research Institute, Vancouver, British Columbia, Canada; b Austrian Institute of Technology, Austria; c Department of Neurology, Medical University of Vienna, Vienna, Austria; d Clinical Research Support Unit, BC Children's Hospital Research Institute, Vancouver, British Columbia, Canada; e Department of Mechanical Engineering, Faculty of Applied Science, University of British Columbia, Vancouver, British Columbia, Canada; f Institute National de la Santé et de la Recherche Médicale (INSERM), Paris, France; g School of Kinesiology, Faculty of Education and Department of Computer Science, Faculty of Science, University of British Columbia, Vancouver, British Columbia, Canada; h Department of Pediatrics, Faculty of Medicine, University of British Columbia, Vancouver, British Columbia, Canada

**Keywords:** Movement disorders, Sleep-related movement disorders, Misdiagnosis, Over-medication, Adverse drug reactions

## Abstract

The cartoon Fidgety Philip, the banner of Western-ADHD diagnosis, depicts a ‘restless’ child exhibiting hyperactive-behaviors with hyper-arousability and/or hypermotor-restlessness (H-behaviors) during sitting. To overcome the gaps between differential diagnostic considerations and modern computing methodologies, we have developed a non-interpretative, neutral pictogram-guided phenotyping language (PG-PL) for describing body-segment movements during sitting (*Journal of Psychiatric Research*). To develop the PG-PL, seven research assistants annotated three original Fidgety Philip cartoons. Their annotations were analyzed with descriptive statistics. To review the PG-PL's performance, the same seven research assistants annotated 12 snapshots with free hand annotations, followed by using the PG-PL, each time in randomized sequence and on two separate occasions. After achieving satisfactory inter-observer agreements, the PG-PL annotation software was used for reviewing videos where the same seven research assistants annotated 12 one-minute long video clips. The video clip annotations were finally used to develop a machine learning algorithm for automated movement detection (*Journal of Psychiatric Research*). These data together demonstrate the value of the PG-PL for manually annotating human movement patterns. Researchers are able to reuse the data and the first version of the machine learning algorithm to further develop and refine the algorithm for differentiating movement patterns.

## Specifications Table

**Table:** 

Subject	Psychiatry and Mental Health
Specific subject area	Annotations of cartoons, pictures, and videos
Type of data	Table Figure Cartoons Images Videos Free-hand annotations Pictogram annotations
How data were acquired	Phase 1: The data were collected using a survey developed in REDCap (Research Electronic Data Capture). The survey can be viewed in Supplementary File 1. Phase 2: The data were collected using custom annotation software (Annotator©, Austrian Institute for Technology, Vienna, Austria).
Data format	Raw Analyzed
Parameters for data collection	Phase 1: Step 1. Cartoon images were obtained from the original Fidgety Philip story for annotation [1]. Phase 1: Step 2. Participants underwent a Suggested Clinical Immobilization Test [2]. The test was recorded on video. Snapshots were taken from the video recordings for annotation.
	Phase 2: One-minute long video clips were taken from the video recordings for annotation.
Description of data collection	Phase 1: Step 1. Research assistants, who were naïve to annotation, annotated the cartoons using free-hand descriptions. Phase 1: Step 2. The same research assistants annotated 12 snapshots using free-hand descriptions and pictograms. Phase 2: The same research assistants annotated 12 one-minute video clips using pictograms.
Data source location	Institution: BC Children's Hospital Research Institute, The University of British Columbia City/Town/Region: Vancouver (British Columbia) Country: Canada
Data accessibility	Repository name: Mendeley Data (for annotation data) Data identification number: https://doi.org/10.17632/ytst4kss9p.5 Direct URL to data: https://data.mendeley.com/datasets/ytst4kss9p/5 Repository name: GitHub (for algorithm source code) Data identification number: https://doi.org/10.5281/zenodo.4382849 Direct URL to data: https://zenodo.org/record/4382849#.X-GB1OlKgfE
Related research article	Beyzaei, N., Bao, S., Bu, Y., Hung, L., Hussaina, H., Maher, K. S., Chan, M., Garn, H., Kloesch, G., Kohn, B., Kuzeljevic, B., McWilliams, S., Spruyt, K., Tse, E., Van der Loos, H.F.M., Kuo, C., Ipsiroglu, O. S. Is Fidgety Philip's ground truth also ours? The creation and application of a machine learning algorithm. J Psychiatr Res. 2020 Dec;131:144-151. https://doi.org/10.1016/j.jpsychires.2020.08.033.

## Value of the Data

• This data demonstrates the utility of the pictogram guided phenotyping language (PG-PL) [3] for manually annotating human movement patterns in video clips, and formed the basis of a machine learning algorithm for automated movement detection.
• Researchers will be able to utilize this data for the further development of the machine learning algorithm that can be used to differentiate restless behaviors.
• The algorithm developed from this data is available at https://doi.org/10.17632/ytst4kss9p.5 [4]. Users can download the algorithm as a standalone application and then use it right away for analyses. We have created a standard operating procedure/readme for using this algorithm (Supplementary File 4). The source code of the algorithm is available at https://doi.org/10.5281/zenodo.4382849 [5] and is provided for software developers who may wish to modify and/or improve the algorithm. To learn more about the motivations underlying the algorithm and the developers of the algorithm, visit the following websites: https://sleepnetwork.org/, http://humbl.bme.ubc.ca/clinical-applications.html, and https://www.bcchr.ca/oipsiroglu.
• The development of a machine learning algorithm using this data will allow in-depth clinical phenotyping of restless behaviors seen in conditions such as Attention Deficit Hyperactivity Disorder, Willis Ekbom disease/restless legs syndrome, and agitation syndrome [6, 7, 8].
• Phenotyping such restless behaviors may enable clinicians to more easily discriminate between the distinct characteristics of the three major restlessness-associated clinical presentations (Attention Deficit Hyperactivity Disorder, Willis Ekbom disease/restless legs syndrome, and agitation syndrome) [6, 7, 8].

## 1 Data Description

This paper describes the collected data and associated statistical analyses for Phase 1 of the study reported in Beyzaei et al. [9]. The data were collected using a survey developed in the REDCap (Research Electronic Data Capture) platform [10, 11], which can be viewed in its entirety in Supplementary File 1. The data can be viewed in Chan et al. [4]. This paper also makes available the video annotation data that laid the foundation for the development of a machine learning algorithm in Phase 2 of the study, which was described in depth by Beyzaei et al. [9].

**Fig. 1:**
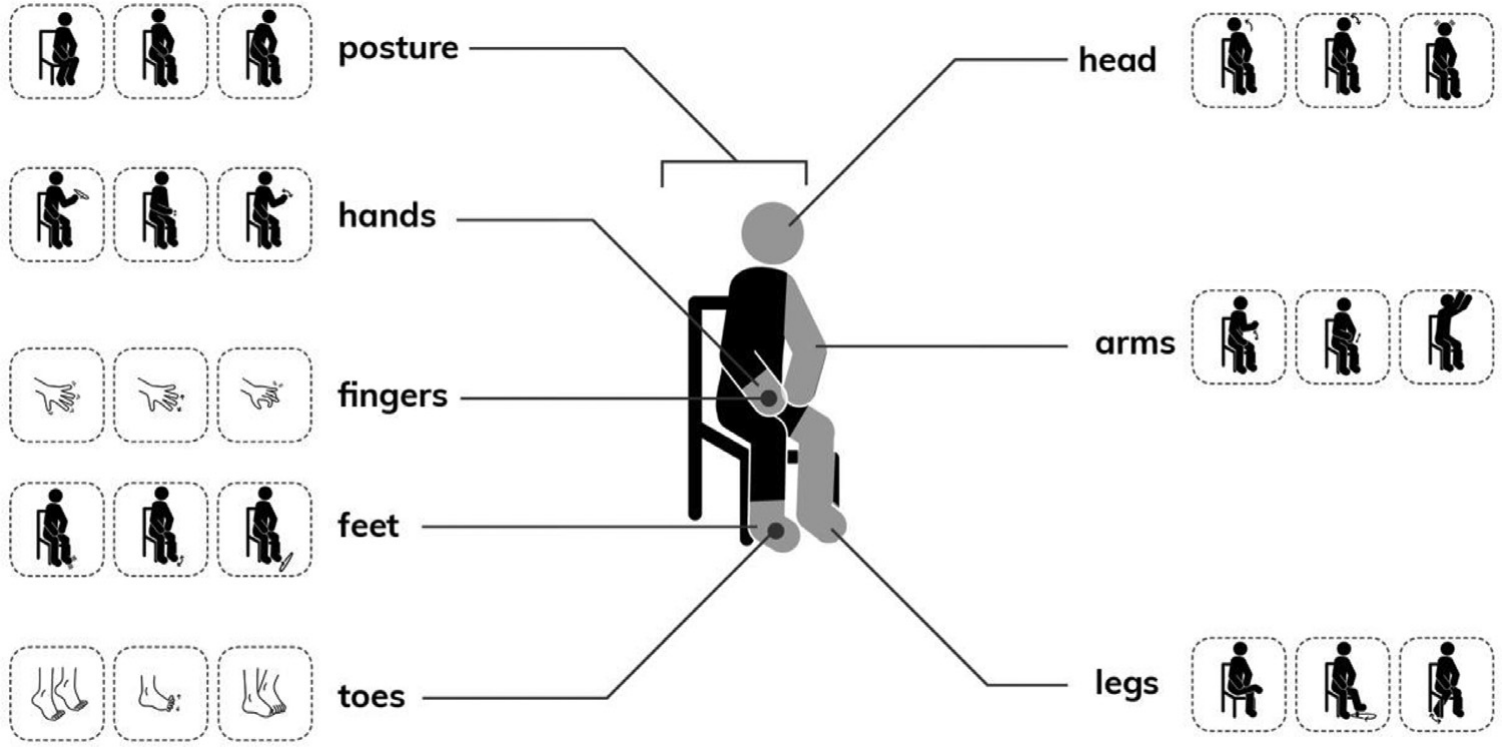
**Pictograms.** Examples of pictograms developed to characterize body movements: posture; head; upper body (comprising arms, hands, fingers); and lower body (comprising legs, feet, toes).

**Phase 1: Step 1.** Supplementary File 2 contains the analysis of the free-hand descriptions of each Fidgety Philip cartoon. Table 1 shows a summary of the average number of free-hand descriptions (neutral vs. interpretive) per cartoon. Fig. 1 shows sample pictograms from the pictogram set.

**Table 1:** **Free-hand Descriptions of Fidgety Philip Cartoons.***Descriptive information for each Fidgety Philip cartoon was separated into neutral and interpretive descriptions.*

Cartoon #	Description	Total	Mean +/- Standard Deviation
1	Neutral	48	6.9 +/- 3.3
	Interpretive	43	6.1 +/- 2.1
2	Neutral	58	8.3 +/- 4.3
	Interpretive	39	5.6 +/- 4.1
3	Neutral	62	8.9 +/- 4.1
	Interpretive	24	3.4 +/- 2.6
Overall	Neutral	168	8 +/- 3.8
	Interpretive	106	5.5 +/- 3.1

**Fig. 2:**
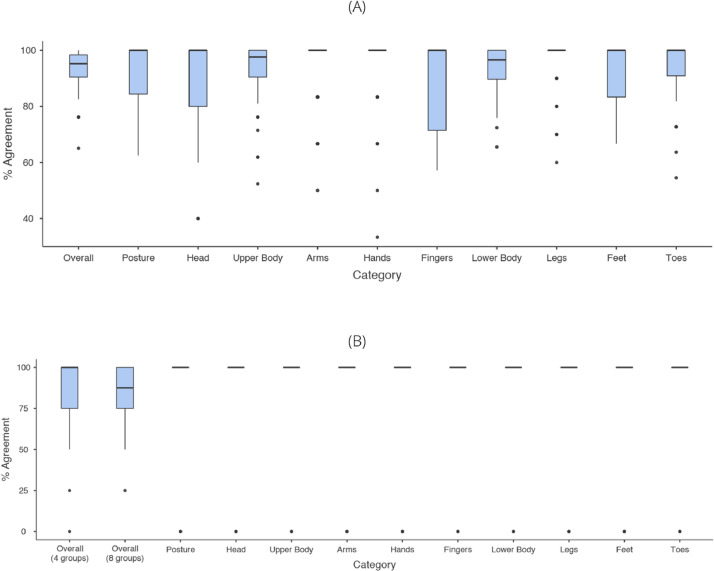
**Intra-Observer Reliability of the Pictogram Guided-Phenotyping Language Before and After Relabeling of Pictograms.** The average percent agreement is shown for each RA utilizing pictograms for describing overall, posture, head, upper body (in detail: arm, hand and finger), and lower body (in detail: legs, feet and toe) movements. RAs' pictogram descriptions on Day 1 were compared to their descriptions on Day 2. (A) Results before relabeling and grouping of pictograms. (B) Results after relabeling and grouping of pictograms. “Overall (4 groups)” refers to reliability when calculated with posture, head, upper body, and lower body. “Overall (8 groups)” refers to reliability when calculated with posture, head, and all detailed categories.

**Phase 1: Step 2.** Supplementary File 3 contains the analysis of the free-hand descriptions of snapshots.  Table 2 shows a summary of the number of free-hand descriptions (neutral vs. interpretive) per day per snapshot. Fig. 2 shows the intra-observer reliability before (A) and after (B) grouping. Table 3 shows the inter-observer reliability before (A) and after (B) grouping.

**Table 2:** **Free-hand Descriptions of Snapshots.***On Day 1 and Day 2, each snapshot was annotated using free-hand descriptions, then reviewed and categorized as descriptive or interpretive, first by the research assistants individually and then by the entire team together as a shared language development exercise. Note the low number of interpretive descriptions on both days, and the increase in mean neutral descriptions (with smaller standard deviations) from Day 1 to Day 2.*

	Day 1						Day 2					
	# of Interpretive Descriptions			# of Neutral Descriptions			# of Interpretive Descriptions			# of Neutral Descriptions		
Snapshot	Mean	Median	Standard Deviation	Mean	Median	Standard Deviation	Mean	Median	Standard Deviation	Mean	Median	Standard Deviation
1	1.29	1	0.95	5.14	4	2.41	1.43	1	0.22	6.86	7	0.53
2	2.14	2	1.21	5.14	4	3.24	2.29	1	0.33	6.43	6	0.44
3	1.57	1	1.27	4.86	5	2.12	1.57	1	0.18	6.29	7	0.68
4	2.00	2	1.15	4.57	4	1.27	2.29	2	0.43	6.71	6	0.59
5	2.14	3	1.46	5.71	6	1.38	1.57	1	0.33	6.43	7	0.50
6	1.29	1	1.11	5.86	6	1.21	1.00	0	0.22	7.14	7	0.66
7	0.57	0	0.79	7.14	7	1.57	1.14	1	0.75	7.71	8	0.68
8	2.00	2	1.41	6.14	6	1.21	2.14	3	0.36	6.14	6	0.60
9	1.29	1	1.50	6.71	6	2.14	1.71	2	0.22	7.43	8	0.68
10	2.43	2	1.90	6.29	6	2.21	1.71	1	0.41	7.29	7	0.70
11	2.14	1	2.41	6.57	6	1.51	2.00	0	0.47	6.86	8	0.61
12	1.43	1	1.13	6.43	7	1.51	1.71	2	0.60	6.29	6	0.69
Overall	1.69	1	1.42	5.88	6	1.94	1.71	1	1.59	6.80	7	1.70

**Table 3:** **Inter-Observer Reliability of the Pictogram Guided-Phenotyping Language Before and After Relabeling and Grouping of Pictograms.***The average percent agreement among all RAs utilizing pictograms for describing overall, posture, head, upper body (in detail: arm, hand and finger) and lower body (in detail: legs, feet and toe) movements on day 1 and day 2. (A) Results before relabeling and grouping of pictograms. (B) Results after relabeling and grouping of pictograms.*

		(A) Before Relabeling and Grouping						(B) After Relabeling and Grouping					
		Day 1			Day 2			Day 1			Day 2		
Category		Average	Min	Max	Average	Min	Max	Average	Min	Max	Average	Min	Max
Overarching													
	Posture	85.22	73.81	100.00	81.84	73.81	90.48	87.30	52.38	100.00	83.33	42.86	100.00
	Head	84.60	61.9	100.00	86.03	58.10	100.00	66.67	42.86	100.00	73.81	42.86	100.00
	Upper Body	90.55	78.68	98.64	91.27	73.70	100.00	78.57	52.38	100.00	71.43	42.86	100.00
	Lower Body	92.17	72.79	95.92	91.69	70.07	100.00	90.48	42.86	100.00	90.48	71.43	100.00

Detailed													
	Arms	93.65	79.64	100.00	93.78	80.30	99.01	80.95	42.86	100.00	83.33	42.86	100.00
	Hands	91.93	73.02	100.00	94.05	68.25	100.00	74.60	42.86	100.00	81.75	42.86	100.00
	Fingers	86.51	73.02	100.00	85.49	74.60	100.00	73.81	42.86	100.00	66.67	42.86	100.00
	Legs	94.52	81.9	100.00	97.22	89.52	100.00	70.64	42.86	100.00	79.37	42.86	100.00
	Feet	89.15	71.43	100.00	89.82	73.02	100.00	78.57	42.86	100.00	80.16	42.86	100.00
	Toes	92.43	82.68	100.00	90.26	74.89	100.00	80.16	42.86	100.00	90.48	71.43	100.00
Overall (All Pictograms)		90.15	81.56	99.09	89.92	79.89	96.52	–	–	–	–	–	–
Overall (Posture, Head & All Detailed Categories)		–	–	–	–	–	–	76.59	59.52	96.43	79.86	57.14	96.43
Overall (Posture, Head, Upper Body & Lower Body)		–	–	–	–	–	–	80.75	57.14	92.86	79.76	57.14	92.86

**Phase 2.** Supplementary File 4 is the machine learning algorithm and associated standard operating procedure developed by and reported in Beyzaei et al. [9].

## 2 Experimental Design, Materials and Methods

**Phase 1: Step 1.** The goal of this step was to train RAs, who were naïve to annotation. Seven undergraduate RAs (median age: 19.5; range 18-21), supervised by two senior faculty members (GK, OSI), annotated three original Fidgety Philip cartoons who were instructed to ‘describe, but not interpret’. The aim was to demonstrate the difference between “neutral, non-interpretative” annotations vs. common interpretative phenomenology in a model setting [12]. ***Data collection.*** RAs separately described each cartoon with a maximum analysis time of 8.5 minutes per cartoon. The order of cartoons was reversed (2-1-3 instead of 1-2-3) to avoid assumptions based on the ongoing sequence and to start a discussion on possible prejudice and/or opinionated views. The data were collected in REDCap (see Supplementary File 1, pp. 2-7) in two categories: (a) descriptions of the current scene, and (b) descriptions predicting what happens next and explaining the prediction. These data can be viewed in Chan et al. [4]. ***Data analysis.*** As a group, RAs first categorized their annotations as “neutral/descriptive” or “interpretive” (generating count data), and RAs noted any negotiation points during their discussions (see [4]). We then descriptively analyzed the count data. The total number of interpretive descriptions was lower than neutral ones, with a trend of decreasing interpretive descriptions per cartoon, despite variations at an individual level (see Table 1 for a summary). ***Transition to Step 2.*** To conclude this step, RAs used pictograms to negotiate the main characteristics in each cartoon and to review to what degree the pictograms could be used for annotation. The pictogram set was created based on parental quotations [2] and utilized 63 pictograms to distinguish between overarching categories of body parts (i.e., posture, head, upper body, lower body) and more detailed categories (i.e., arms, hands, fingers, legs, feet, toes) [3] (see Fig. 1). In a roundtable, the group agreed that pictograms will reduce challenges associated with free-hand annotations (mainly different connotations of wordings) and streamline the annotation process.

**Phase 1: Step 2.** The goal of this step was to develop a shared language based on the pictogram set (now termed the PG-PL). To work out the distinction between “neutral/descriptive” and “interpretive” PG-PL descriptions, the same RAs in Phase 1, Step 1, were tasked with annotating snapshots of human volunteers. ***Materials.*** Healthy, young adult volunteers (n=18; 10 females; age range: 19-26 years) visited our laboratory and individually underwent a Suggested Clinical Immobilization Test [2]. Each Test was recorded on video. We randomly selected video stills (i.e., snapshots) from the recordings for annotation by the RAs. ***Data collection.*** RAs first annotated 12 snapshots using free-hand descriptions and then the PG-PL. Two days later, the RAs repeated the exercise using a randomized viewing order. The data were collected in REDCap (see Supplementary File 1, pp. 8-33). These data can be viewed in Chan et al. [4]. ***Data analysis.*** First, RAs categorized their free-hand annotations as “neutral/descriptive” or “interpretive,” which we then descriptively analyzed (Supplementary File 2). We found that there were fewer interpretive and more descriptive free-hand annotations on both days (see Table 2 for a summary). We then computed intra-observer reliability and inter-observer reliability on all 63 pictograms to determine agreement on PG-PL use (Fig. 2A, Table 3A). However, we realized that this calculation included all characteristics (including not observed), which likely caused high agreement since it is not possible to observe all pictograms simultaneously (e.g., RAs could only select one out of eight posture pictograms and the subject cannot be both “sitting straight” and “sitting hunched over”). Therefore, to reduce the number of choices and only highlight the movement without describing its characteristics, which were seen as interpretive, we grouped the pictograms into eight categories (posture, head, arms, and fingers into an “upper body” category, and legs, feet, toes into a “lower body” category to be confident that there was no misinterpretation of body parts. Groupings resulted in either “movement: yes” or “movement: no” for each category. We then recomputed intra-observer reliability and inter-observer reliability (Fig. 2B, Table 3B). ***Transition to Phase 2.*** After achieving satisfactory average intra-observer reliability and inter-observer reliability (approximately 80%), we proceeded to Phase 2.

**Phase 2.** The goal of this phase was to apply the PG-PL to SCIT videos and to develop the first machine learning algorithm for automated movement detection [9]. The reader is referred to our main paper [9] for more details on this phase. We present here the dataset that was foundational to this phase. ***Materials.*** From the same video recordings in Phase 1, Step 2, we randomly selected one-minute long video clips for annotation by the RAs. ***Data.*** As described in the main paper [9], the same RAs annotated 12 one-minute long video clips using the custom annotation software, Annotator© (Austrian Institute for Technology, Vienna, Austria). These data can be viewed in Chan et al. [4]. These data are available in a raw format (i.e., direct output from Annotator) and in a processed format (which was necessary for calculating inter-observer reliability as reported in [9, Fig. 2]). The raw format is not appropriate for inter-observer reliability analysis, as there is no standardization. Therefore, we used a computer script to re-code the data into epochs, which enabled us to calculate inter-observer reliability for each epoch and average across all epochs [9]. ***Machine learning algorithm.*** The standard operating procedure/readme file of the machine learning algorithm that was developed based on the video annotation data can be found in Supplementary File 4.

## Ethics Statement

This study was approved by The University of British Columbia's Clinical Research Ethics Board (H15-01090). In accordance with the Declaration of Helsinki, informed consent was obtained from the human subjects who provided the videos.

## CRediT Author Statement

**Melvin Chan:** Formal analysis, Writing - Review & Editing; **Emmanuel K. Tse:** Formal analysis; **Nadia Beyzaei:** Resources; **Seraph Bao:** Formal analysis; **Yanyun Bu:** Software; **Linus Hung:** Software; **Hebah Hussaina:** Formal analysis; **Khaola Safia Maher:** Formal analysis; **Heinrich Garn:** Methodology, Software; **Gerhard Kloesch:** Methodology; **Bernhard Kohn:** Methodology, Software; **Boris Kuzeljevic:** Formal analysis; **Scout McWilliams:** Formal analysis, Writing - Review & Editing; **Karen Spruyt:** Methodology; **Hendrik F. Machiel Van der Loos:** Writing - Review & Editing, Supervision, Funding Acquisition; **Calvin Kuo:** Writing - Review & Editing, Supervision; **Osman Ipsiroglu:** Conceptualization, Writing - Original Draft, Writing - Review & Editing, Supervision, Funding Acquisition

## Declaration of Competing Interest

The authors declare that they have no known competing financial interests or personal relationships which have, or could be perceived to have, influenced the work reported in this article.

## Appendix Supplementary materials

Image, application 1

Image, application 2

Image, application 3

Image, application 4

## References

[1] Hoffman H. (1845). Funny stories and droll pictures with 15 beautifully colored plates for children ages 3 to 6. Literarische Anstalt.

[2] Ipsiroglu O.S., Beyzaei N., Berger M., Wagner A.L., Dhalla S., Garden J., Stockler S. (2016). Emplotted narratives” and structured “behavioral observations” supporting the diagnosis of Willis-Ekbom disease/restless legs syndrome in children with neurodevelopmental conditions. CNS Neurosci. Ther..

[3] Beyzaei N., Bao S., Maher S., Silvestri R., Walters A., Dorffner G., Kloesch G., Spruyt K., Ipsiroglu O.S. (2019). Using pictograms to make “structured behavioral observations” of youth with restless legs syndrome reproducible. Sleep Med..

[4] M. Chan, E. Tse, S. Bao, M. Berger, N. Beyzaei, M. Campbell, H. Garn, H. Hussaina, G. Kloesch, B. Kohn, B. Kuzeljevic, Y. J. Lee, K. S. Maher, N. Carson, J. Jeyaratnam, S. McWilliams, K. Spruyt, H.F.M. Van der Loos, C. Kuo, and O. S. Ipsiroglu, Fidgety Philip and the Suggested Clinical Immobilization Test: Annotation dataset & HMovements automated movement detection algorithm, Mendeley Data, V5, 2020. https://doi.rg/10.17632/ytst4kss9p.5.

[5] Hung, L. & Kuo, C. HMovements code publication release. 2020 Dec 21. https://doi.org/10.5281/zenodo.4382849.

[6] Walters A.S., Silvestri R., Zucconi M., Chandrashekariah R., Konofal E. (2008). Review of the possible relationship and hypothetical links between attention deficit hyperactivity disorder (ADHD) and the simple sleep related movement disorders, parasomnias, hypersomnias, and circadian rhythm disorders. J. Clin. Sleep Med..

[7] Allen R.P., Picchietti D.L., Garcia-Borreguero D., Ondo W.G., Walters A.S., Winkelman J.W., Zucconi M., Ferri R., Trenkwalder C., Lee H.B (2014). Restless legs syndrome/Willis–Ekbom disease diagnostic criteria: Updated International Restless Legs Syndrome Study Group (IRLSSG) consensus criteria – History, rationale, description, and significance. Sleep Med..

[8] Luft M.J., Lamy M., DelBello M.P., McNamara R.K., Strawn J.R. (2018). Antidepressant-induced activation in children and adolescents: Risk, recognition and management. Curr. Probl. Pediatr. Adolesc. Health Care..

[9] Beyzaei N., Bao S., Bu Y., Hung L., Hussaina H., Maher K.S., Chan M., Garn H., Kloesch G., Kohn B., Kuzeljevic B., McWilliams S., Spruyt K., Tse E., Van der Loos H.F.M., Kuo C., Ipsiroglu O.S (2020). Is Fidgety Philip's ground truth also ours? The creation and application of a machine learning algorithm. J. Psychiatr. Res..

[10] Harris P.A., Taylor R., Thielke R., Payne J., Gonzalez N., Conde J.G. (2009). Research electronic data capture (REDCap) – a metadata-driven methodology and workflow process for providing translational research informatics support. J. Biomed. Inform..

[11] Harris P.A., Taylor R., Minor B.L., Elliott V., Fernandez M., O'Neal L., McLeod L., Delacqua G., Delacqua F., Kirby J., Duda S.N. (2019). & REDCap Consortium. The REDCap consortium: Building an international community of software partners. J. Biomed. Inform..

[12] Smith, D. W., Phenomenology. The Stanford encyclopedia of philosophy (Summer 2018 Edition). https://plato.stanford.edu/archives/sum2018/entries/phenomenology/. (Accessed 2 March 2020).

