# Supplementary material for: Fidgety Philip and the Suggested Clinical Immobilization Test: Annotation data for developing a machine learning algorithm
Source: Data Brief. 2021 Jan 17;35:106770. doi: 10.1016/j.dib.2021.106770 (PMC7851356; doi:10.1016/j.dib.2021.106770)
Supplement: Supplementary file 1 [file mmc1.pdf]

Training Module 1: Zappelphilipp Illustrations

Let us know who you are. (Note: this information will only be used to identify your survey responses within our Sleep/Wake-Behaviour Research Lab Setting and will not be shared with any third party groups)

First name: ________________________________

Last name: ________________________________

BCCHR Email Address: ________________________________

Welcome to Training Module One!

This module is intended to be an introduction to our pattern analysis project. By completing this module, you will learn to make structured observations and create a hierarchy of the main characteristics you observe in illustrations.

You are an integral part in this project, so please fill out the survey below in as much detail as possible!

Zappelphillip Illustration 1

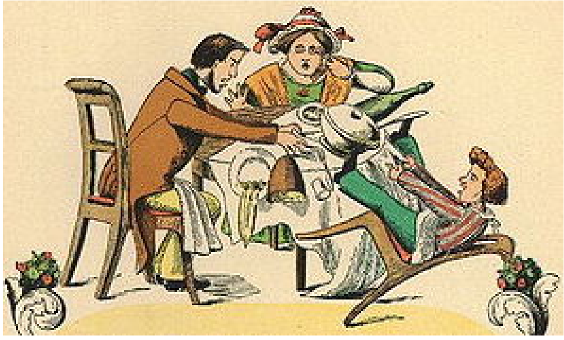

'Let me see if Philip can / Be a little gentleman; / Let me see if he is able / To sit still for once at the table.' / Thus spoke, in earnest tone, / The father to his son; / And the mother looked very grave / To see Philip so misbehave.

Describe everything you see in the Zappelphillip illustration. __________________________________

Create a hierarchy: Rank your initial descriptions in the order you observed them. You may copy and paste your descriptions from the field above and add additional details as necessary.

1 ____________________________

2 ____________________________

3 ____________________________

4 ____________________________

5 ____________________________

6 ____________________________

7 ____________________________

8 ____________________________

9 ____________________________

10 ____________________________

11 ____________________________

12 ____________________________

13 ____________________________

14 ____________________________

15 ____________________________

16 ______________________

17 ______________________

18 ______________________

19 ______________________

20 ______________________

21 ______________________

22 ______________________

23 ______________________

24 ______________________

25 ______________________

26 ______________________

27 ______________________

28 ______________________

29 ______________________

30 ______________________

List any additional descriptions in the space provided.

____
(Use numerals to separate descriptions (31., 32., ...))

Zappelphillip Illustration 1

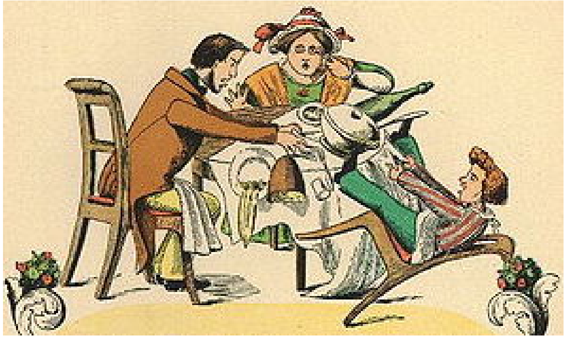

The Zappelphillip illustration, shown again for convenience, is a snapshot in time. Describe what you would expect to happen next.

____

Zappelphillip Illustration 2

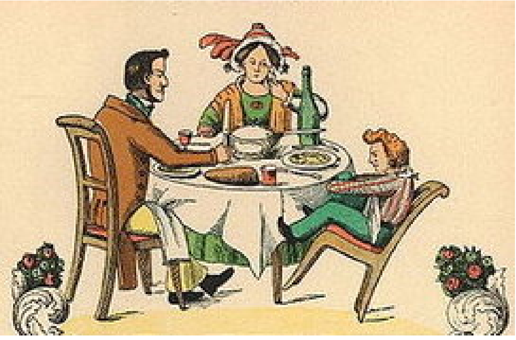

Does the Zappelphillip illustration match what you expected? ◯ Yes ◯ No

Please describe. (e.g. did anything surprise you? would you change any aspect of the illustration to be more inline with what you expected?) __________________________________

Please describe the discrepancies between what you see and what you expected. __________________________________

Describe everything you see in the Zappelphillip illustration. __________________________________

Create a hierarchy: Rank your initial descriptions in the order you observed them. You may copy and paste your descriptions from the field above and add additional details as necessary.

1 ____________________________
2 ____________________________
3 ____________________________
4 ____________________________
5 ____________________________
6 ____________________________
7 ____________________________
8 ____________________________
9 ____________________________
10 ____________________________
11 ____________________________
12 ____________________________
13 ____________________________
14 ____________________________
15 ____________________________

16 __________________________________

17 __________________________________

18 __________________________________

19 __________________________________

20 __________________________________

21 __________________________________

22 __________________________________

23 __________________________________

24 __________________________________

25 __________________________________

26 __________________________________

27 __________________________________

28 __________________________________

29 __________________________________

30 __________________________________

List any additional descriptions in the space provided.

____
(Use numerals to separate descriptions (31., 32., ...))

Zappelphillip Illustration 2

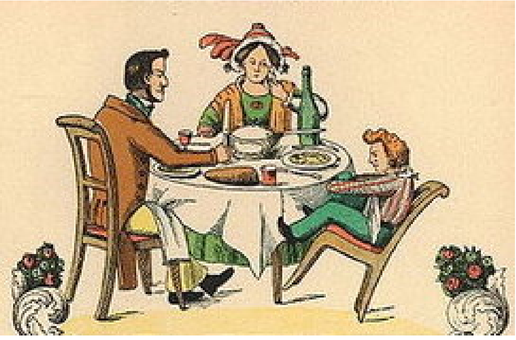

The Zappelphillip illustration, shown again for convenience, is a snapshot in time. Describe what you would expect to happen next.

____

Zappelphillip Illustration 3

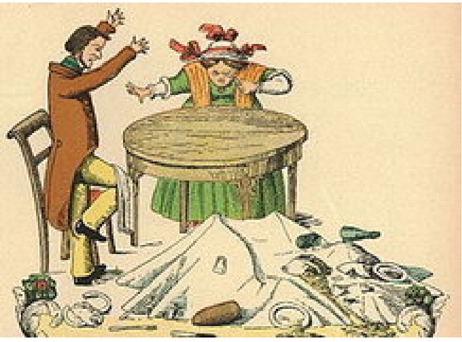

Does the Zappelphillip illustration match what you expected?

◯ Yes
◯ No

Please describe. (e.g. did anything surprise you? would you change any aspect of the illustration to be more inline with what you expected?)

____

Please describe the discrepancies between what you see and what you expected.

____

Describe everything you see in the Zappelphillip illustration.

____

Create a hierarchy: Rank your initial descriptions in the order you observed them. You may copy and paste your descriptions from the field above and add additional details as necessary.

1 __________________________________

2 __________________________________

3 __________________________________

4 __________________________________

5 __________________________________

6 __________________________________

7 __________________________________

8 __________________________________

9 __________________________________

10 __________________________________

11 __________________________________

12 __________________________________

13 __________________________________

14 __________________________________

15 __________________________________

16 ______________________________

17 ______________________________

18 ______________________________

19 ______________________________

20 ______________________________

21 ______________________________

22 ______________________________

23 ______________________________

24 ______________________________

25 ______________________________

26 ______________________________

27 ______________________________

28 ______________________________

29 ______________________________

30 ______________________________

List any additional descriptions in the space provided. ______________________________

Zappelphillip Illustration 3

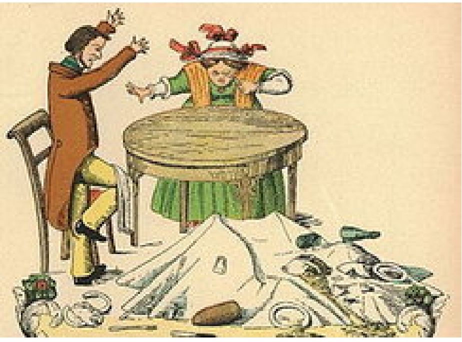

The Zappelphillip illustration, shown again for convenience, is a snapshot in time. Describe what you would expect to happen next. ______________________________

Training Module 2: SCIT Snapshots

Welcome to Training Module Two!

By completing this module, you will learn to make structured observations and create a hierarchy of the main characteristics you observe in snapshot images.

You are an integral part in this project, so please fill out the survey below in as much detail as possible!

SCIT Snapshot 1

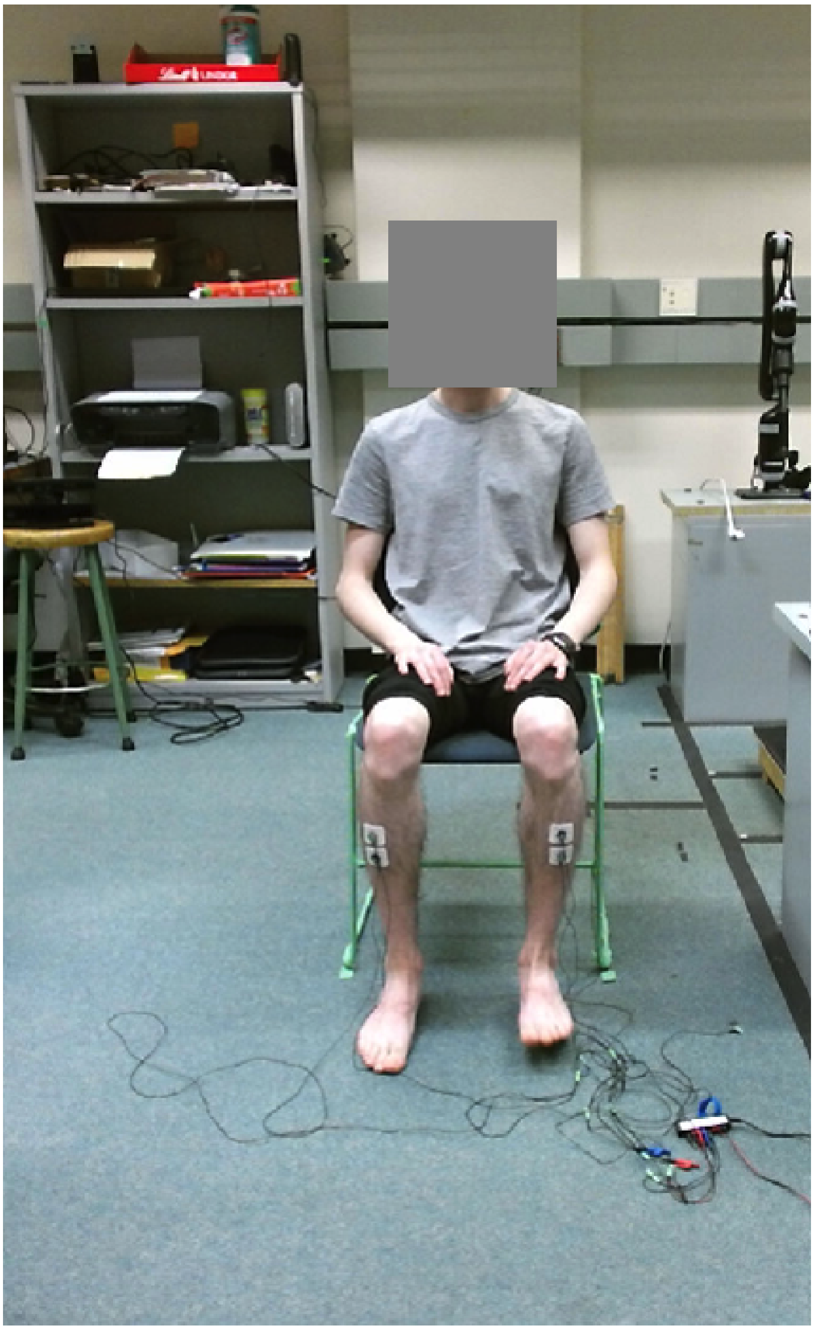

Describe everything you see in the snapshot.

____

Create a hierarchy: Rank your initial descriptions in the order you observed them. You may copy and paste your descriptions from the field above and add additional details as necessary.

1 ______________________________

2 ______________________________

3 ______________________________

4 ______________________________

5 ______________________________

6 ______________________________

7 ______________________________

8 ______________________________

9 ______________________________

10 ______________________________

11 ______________________________

12 ______________________________

13 ______________________________

14 ______________________________

15 ______________________________

16 ______________________________

17 ______________________________

18 ______________________________

19 ______________________________

20 ______________________________

21 ______________________________

22 ______________________________

23 ______________________________

24 ______________________________

25 ______________________________

26 ______________________________

27 ______________________________

28 ______________________________

29 ______________________________

30 __________________________________

List any additional descriptions in the space provided.

____
(Use numerals to separate descriptions (31., 32., ...))

This image is a snapshot in time. Describe what you would expect to happen next.

____

SCIT Snapshot 2

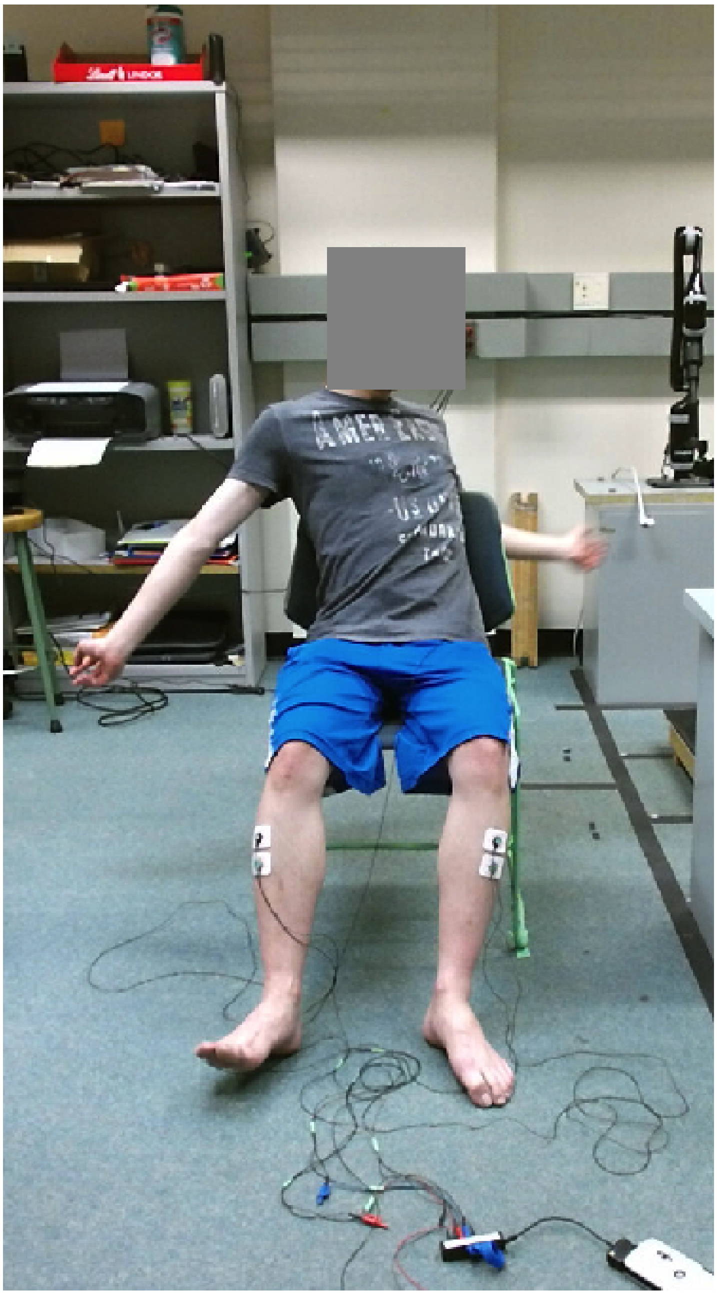

Describe everything you see in the snapshot. __________________________________

Create a hierarchy: Rank your initial descriptions in the order you observed them. You may copy and paste your descriptions from the field above and add additional details as necessary.

1 ____________________________

2 ____________________________

3 ____________________________

4 __________________________________

5 __________________________________

6 __________________________________

7 __________________________________

8 __________________________________

9 __________________________________

10 __________________________________

11 __________________________________

12 __________________________________

13 __________________________________

14 __________________________________

15 __________________________________

16 __________________________________

17 __________________________________

18 __________________________________

19 __________________________________

20 __________________________________

21 __________________________________

22 __________________________________

23 __________________________________

24 __________________________________

25 __________________________________

26 __________________________________

27 __________________________________

28 __________________________________

29 __________________________________

30 __________________________________

List any additional descriptions in the space provided. __________________________________
(Use numerals to separate descriptions (31., 32., ...))

This image is a snapshot in time. Describe what you would expect to happen next. __________________________________

SCIT Snapshot 3

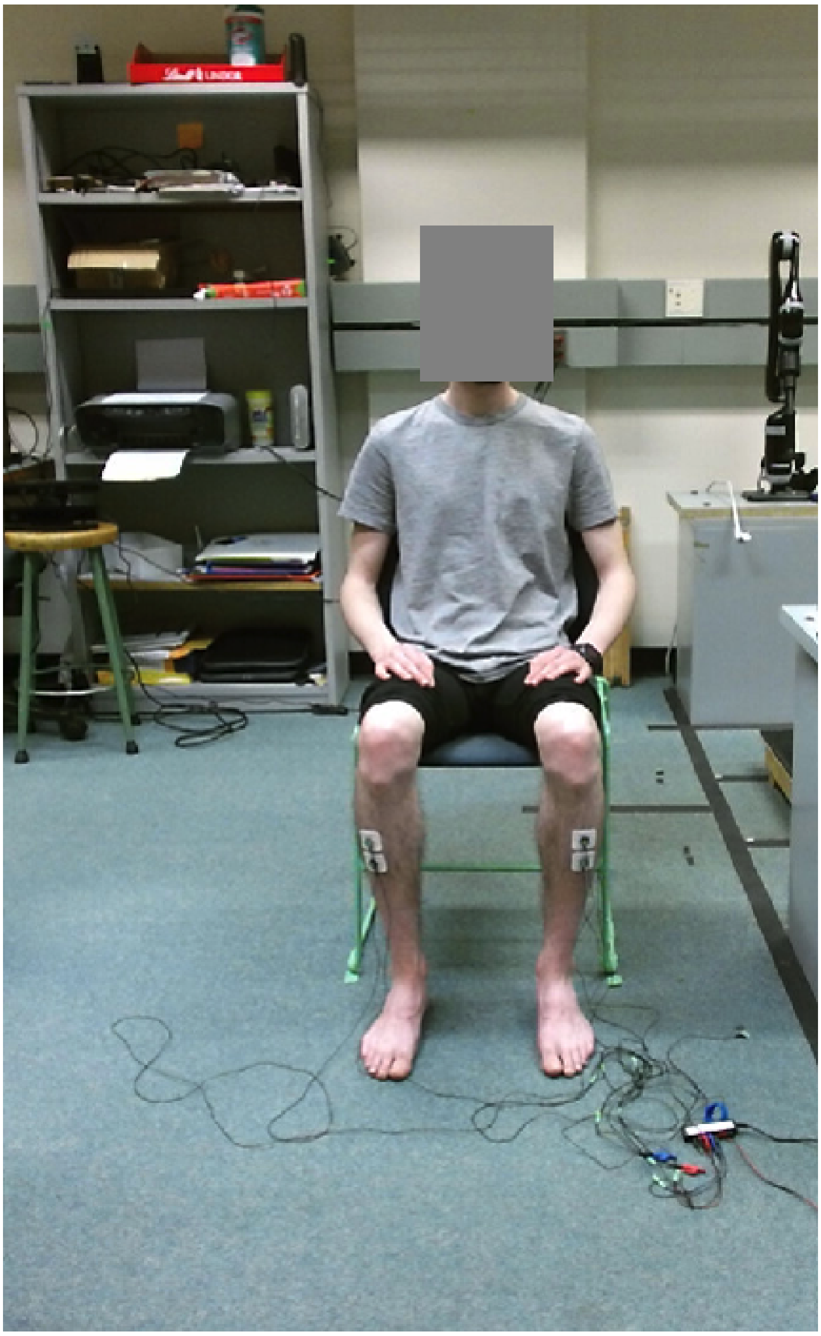

Describe everything you see in the snapshot.

Create a hierarchy: Rank your initial descriptions in the order you observed them. You may copy and paste your descriptions from the field above and add additional details as necessary.

1

2

3

4 __________________________________

5 __________________________________

6 __________________________________

7 __________________________________

8 __________________________________

9 __________________________________

10 __________________________________

11 __________________________________

12 __________________________________

13 __________________________________

14 __________________________________

15 __________________________________

16 __________________________________

17 __________________________________

18 __________________________________

19 __________________________________

20 __________________________________

21 __________________________________

22 __________________________________

23 __________________________________

24 __________________________________

25 __________________________________

26 __________________________________

27 __________________________________

28 __________________________________

29 __________________________________

30 __________________________________

List any additional descriptions in the space provided. ____
(Use numerals to separate descriptions (31., 32., ...))

This image is a snapshot in time. Describe what you would expect to happen next. ____

SCIT Snapshot 4

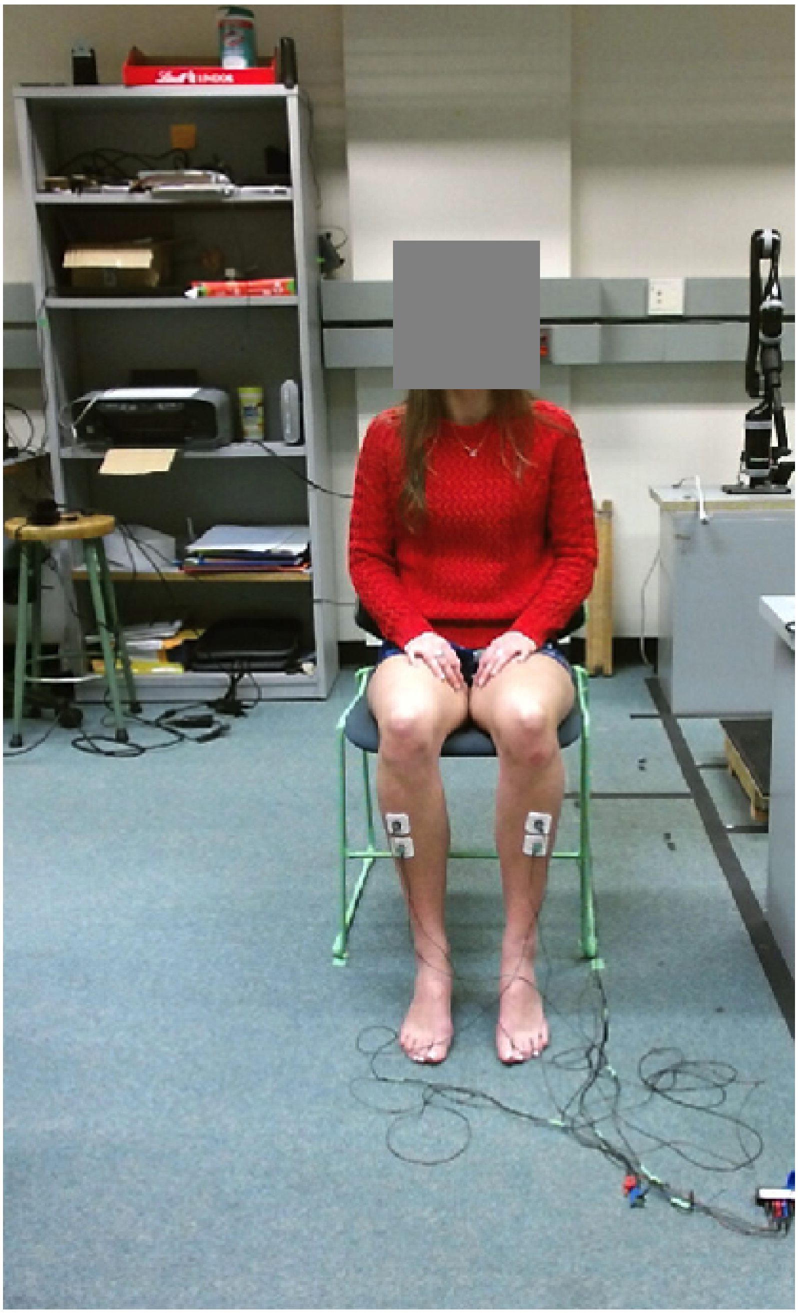

Describe everything you see in the snapshot.

____

Create a hierarchy: Rank your initial descriptions in the order you observed them. You may copy and paste your descriptions from the field above and add additional details as necessary.

1 __________________________________

2 __________________________________

3 __________________________________

4 __________________________________

5 __________________________________

6 __________________________________

7 __________________________________

8 __________________________________

9 __________________________________

10 __________________________________

11 __________________________________

12 __________________________________

13 __________________________________

14 __________________________________

15 __________________________________

16 __________________________________

17 __________________________________

18 __________________________________

19 __________________________________

20 __________________________________

21 __________________________________

22 __________________________________

23 __________________________________

24 __________________________________

25 __________________________________

26 __________________________________

27 __________________________________

28 __________________________________

29 __________________________________

30 __________________________________

List any additional descriptions in the space provided. ____

(Use numerals to separate descriptions (31., 32., ...))

This image is a snapshot in time. Describe what you would expect to happen next. ____

SCIT Snapshot 5

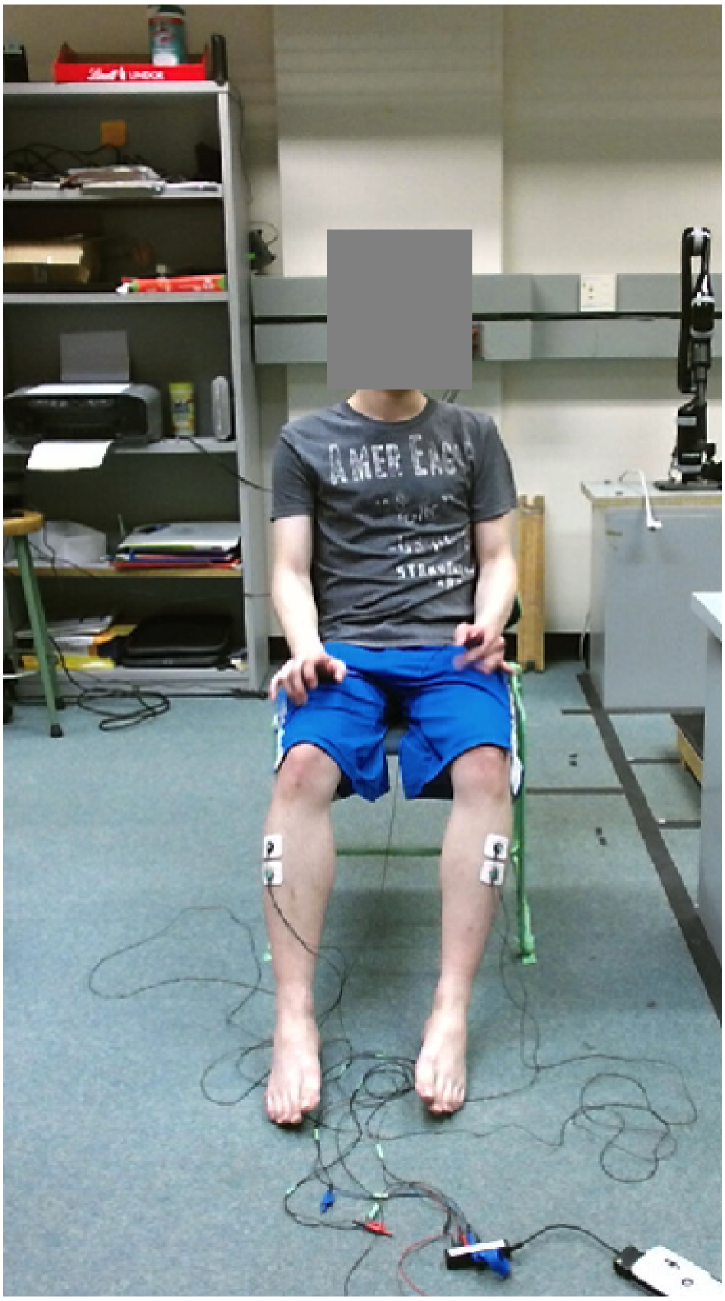

Describe everything you see in the snapshot.

____

Create a hierarchy: Rank your initial descriptions in the order you observed them. You may copy and paste your descriptions from the field above and add additional details as necessary.

1 ________________________________

2 ________________________________

3 ________________________________

4 ______________________

5 ______________________

6 ______________________

7 ______________________

8 ______________________

9 ______________________

10 ______________________

11 ______________________

12 ______________________

13 ______________________

14 ______________________

15 ______________________

16 ______________________

17 ______________________

18 ______________________

19 ______________________

20 ______________________

21 ______________________

22 ______________________

23 ______________________

24 ______________________

25 ______________________

26 ______________________

27 ______________________

28 ______________________

29 ______________________

30 ______________________

List any additional descriptions in the space provided. ______________________
(Use numerals to separate descriptions (31., 32., ...))

This image is a snapshot in time. Describe what you would expect to happen next. ______________________

SCIT Snapshot 6

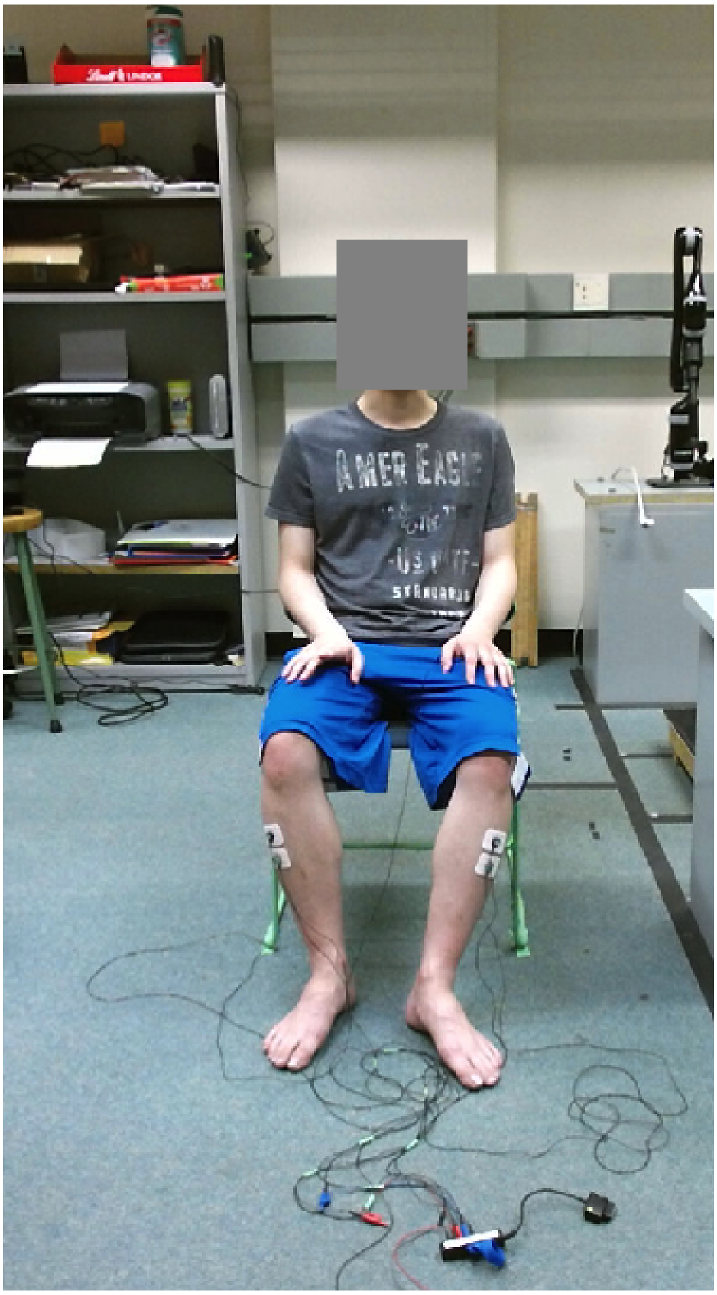

Describe everything you see in the snapshot.

____

Create a hierarchy: Rank your initial descriptions in the order you observed them. You may copy and paste your descriptions from the field above and add additional details as necessary.

1 __________________________________

2 __________________________________

3 __________________________________

4 __________________________________

5 __________________________________

6 __________________________________

7 __________________________________

8 __________________________________

9 __________________________________

10 __________________________________

11 __________________________________

12 __________________________________

13 __________________________________

14 __________________________________

15 __________________________________

16 __________________________________

17 __________________________________

18 __________________________________

19 __________________________________

20 __________________________________

21 __________________________________

22 __________________________________

23 __________________________________

24 __________________________________

25 __________________________________

26 __________________________________

27 __________________________________

28 __________________________________

29 __________________________________

30 __________________________________

List any additional descriptions in the space provided.

____
(Use numerals to separate descriptions (31., 32., ...))

This image is a snapshot in time. Describe what you would expect to happen next.

____

SCIT Snapshot 7

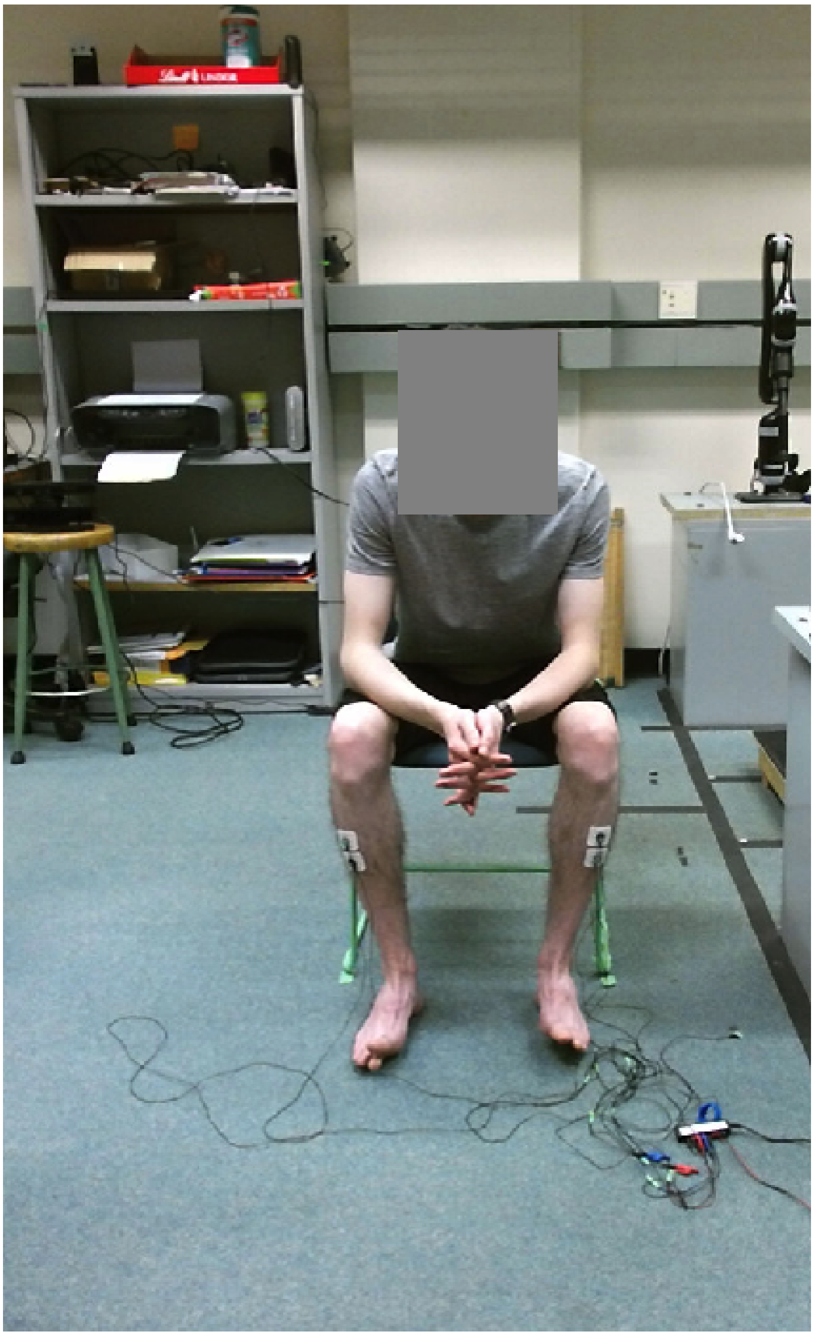

Describe everything you see in the snapshot.

Create a hierarchy: Rank your initial descriptions in the order you observed them. You may copy and paste your descriptions from the field above and add additional details as necessary.

1

2

3

4 __________________________________

5 __________________________________

6 __________________________________

7 __________________________________

8 __________________________________

9 __________________________________

10 __________________________________

11 __________________________________

12 __________________________________

13 __________________________________

14 __________________________________

15 __________________________________

16 __________________________________

17 __________________________________

18 __________________________________

19 __________________________________

20 __________________________________

21 __________________________________

22 __________________________________

23 __________________________________

24 __________________________________

25 __________________________________

26 __________________________________

27 __________________________________

28 __________________________________

29 __________________________________

30 __________________________________

List any additional descriptions in the space provided.

____
(Use numerals to separate descriptions (31., 32., ...))

This image is a snapshot in time. Describe what you would expect to happen next.

____

SCIT Snapshot 8

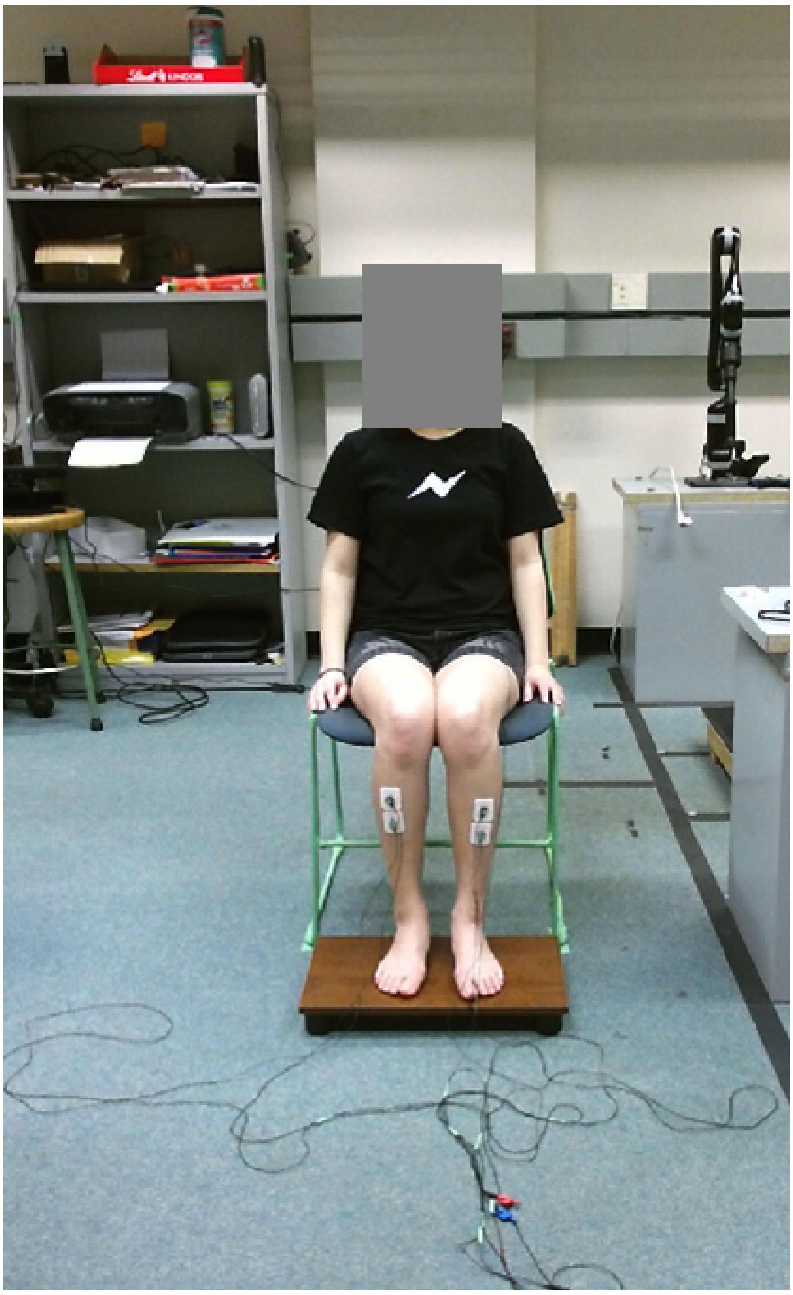

Describe everything you see in the snapshot.

____

Create a hierarchy: Rank your initial descriptions in the order you observed them. You may copy and paste your descriptions from the field above and add additional details as necessary.

1 ____________________________

2 ____________________________

3 ____________________________

4 ______________________________

5 ______________________________

6 ______________________________

7 ______________________________

8 ______________________________

9 ______________________________

10 ______________________________

11 ______________________________

12 ______________________________

13 ______________________________

14 ______________________________

15 ______________________________

16 ______________________________

17 ______________________________

18 ______________________________

19 ______________________________

20 ______________________________

21 ______________________________

22 ______________________________

23 ______________________________

24 ______________________________

25 ______________________________

26 ______________________________

27 ______________________________

28 ______________________________

29 ______________________________

30 ______________________________

List any additional descriptions in the space provided.

____
(Use numerals to separate descriptions (31., 32., ...))

This image is a snapshot in time. Describe what you would expect to happen next.

____

SCIT Snapshot 9

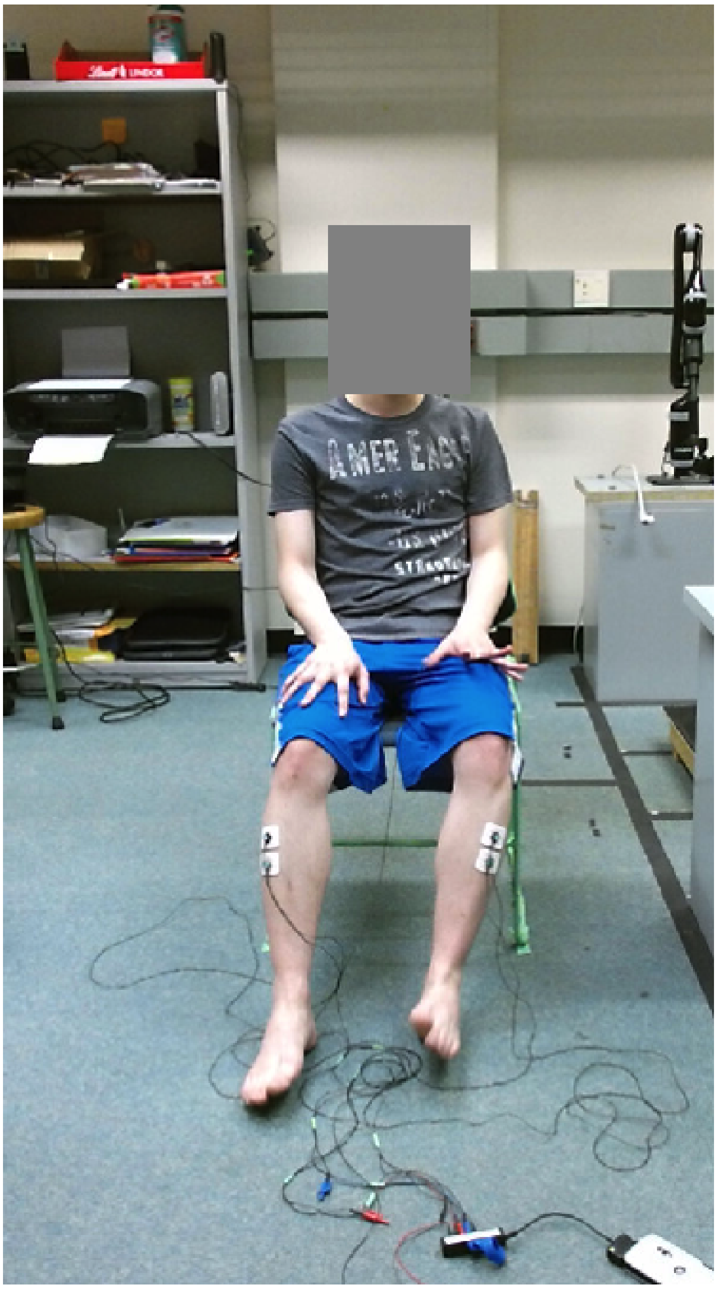

Describe everything you see in the snapshot.

____

Create a hierarchy: Rank your initial descriptions in the order you observed them. You may copy and paste your descriptions from the field above and add additional details as necessary.

1 ____________________________

2 ____________________________

3 ____________________________

4 ____________________________

5 __________________________________

6 __________________________________

7 __________________________________

8 __________________________________

9 __________________________________

10 __________________________________

11 __________________________________

12 __________________________________

13 __________________________________

14 __________________________________

15 __________________________________

16 __________________________________

17 __________________________________

18 __________________________________

19 __________________________________

20 __________________________________

21 __________________________________

22 __________________________________

23 __________________________________

24 __________________________________

25 __________________________________

26 __________________________________

27 __________________________________

28 __________________________________

29 __________________________________

30 __________________________________

List any additional descriptions in the space provided.

____
(Use numerals to separate descriptions (31., 32., ...))

This image is a snapshot in time. Describe what you would expect to happen next.

____

SCIT Snapshot 10

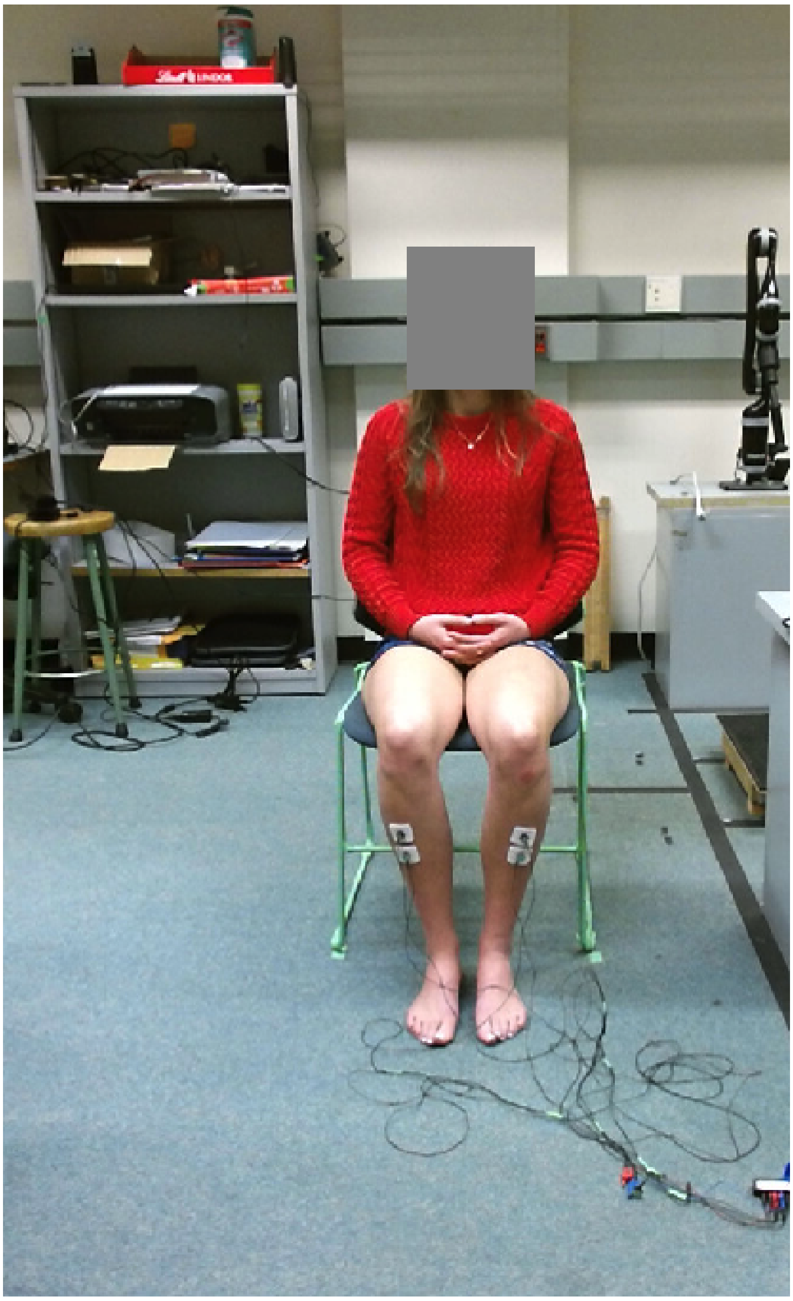

Describe everything you see in the snapshot.

____

Create a hierarchy: Rank your initial descriptions in the order you observed them. You may copy and paste your descriptions from the field above and add additional details as necessary.

1 ________________________________

2 ________________________________

3 ________________________________

4 __________________________________

5 __________________________________

6 __________________________________

7 __________________________________

8 __________________________________

9 __________________________________

10 __________________________________

11 __________________________________

12 __________________________________

13 __________________________________

14 __________________________________

15 __________________________________

16 __________________________________

17 __________________________________

18 __________________________________

19 __________________________________

20 __________________________________

21 __________________________________

22 __________________________________

23 __________________________________

24 __________________________________

25 __________________________________

26 __________________________________

27 __________________________________

28 __________________________________

29 __________________________________

30 __________________________________

List any additional descriptions in the space provided.

____
(Use numerals to separate descriptions (31., 32., ...))

This image is a snapshot in time. Describe what you would expect to happen next.

____

SCIT Snapshot 11

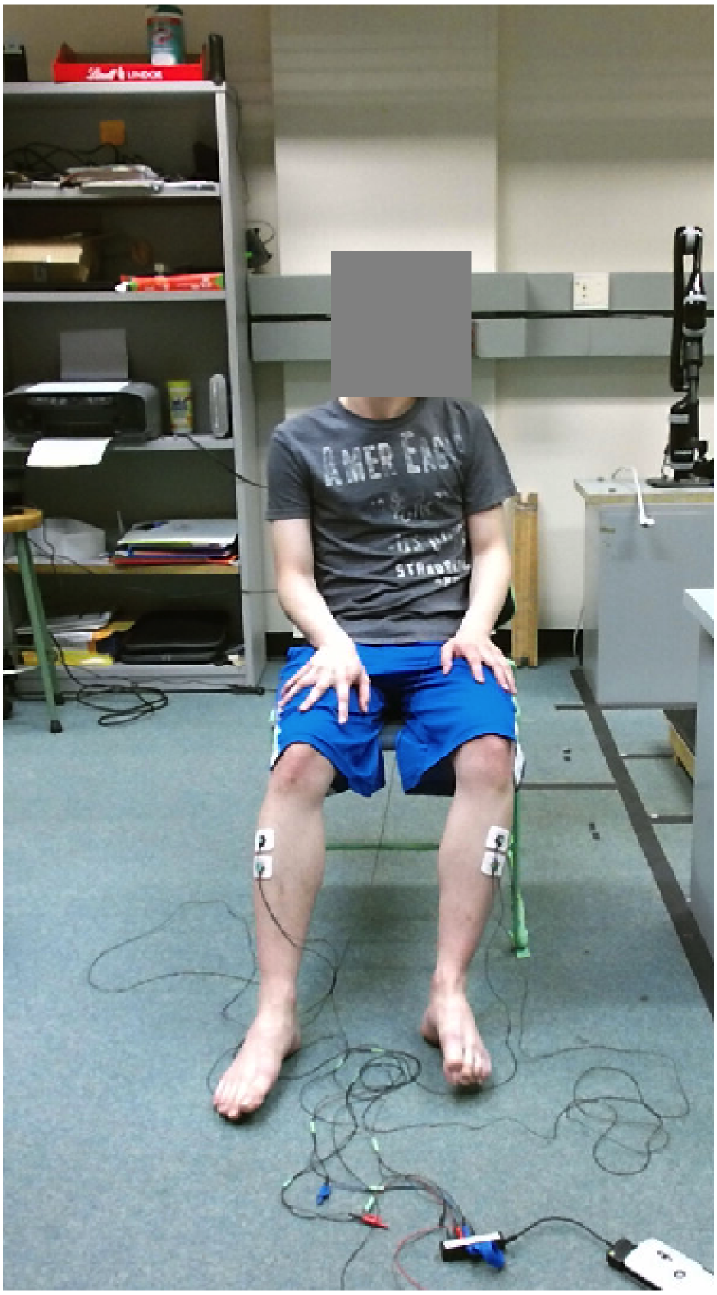

Describe everything you see in the snapshot.

____

Create a hierarchy: Rank your initial descriptions in the order you observed them. You may copy and paste your descriptions from the field above and add additional details as necessary.

1 ______________________________

2 ______________________________

3 ______________________________

4 __________________________________

5 __________________________________

6 __________________________________

7 __________________________________

8 __________________________________

9 __________________________________

10 __________________________________

11 __________________________________

12 __________________________________

13 __________________________________

14 __________________________________

15 __________________________________

16 __________________________________

17 __________________________________

18 __________________________________

19 __________________________________

20 __________________________________

21 __________________________________

22 __________________________________

23 __________________________________

24 __________________________________

25 __________________________________

26 __________________________________

27 __________________________________

28 __________________________________

29 __________________________________

30 __________________________________

List any additional descriptions in the space provided.

____
(Use numerals to separate descriptions (31., 32., ...))

This image is a snapshot in time. Describe what you would expect to happen next.

____

SCIT Snapshot 12

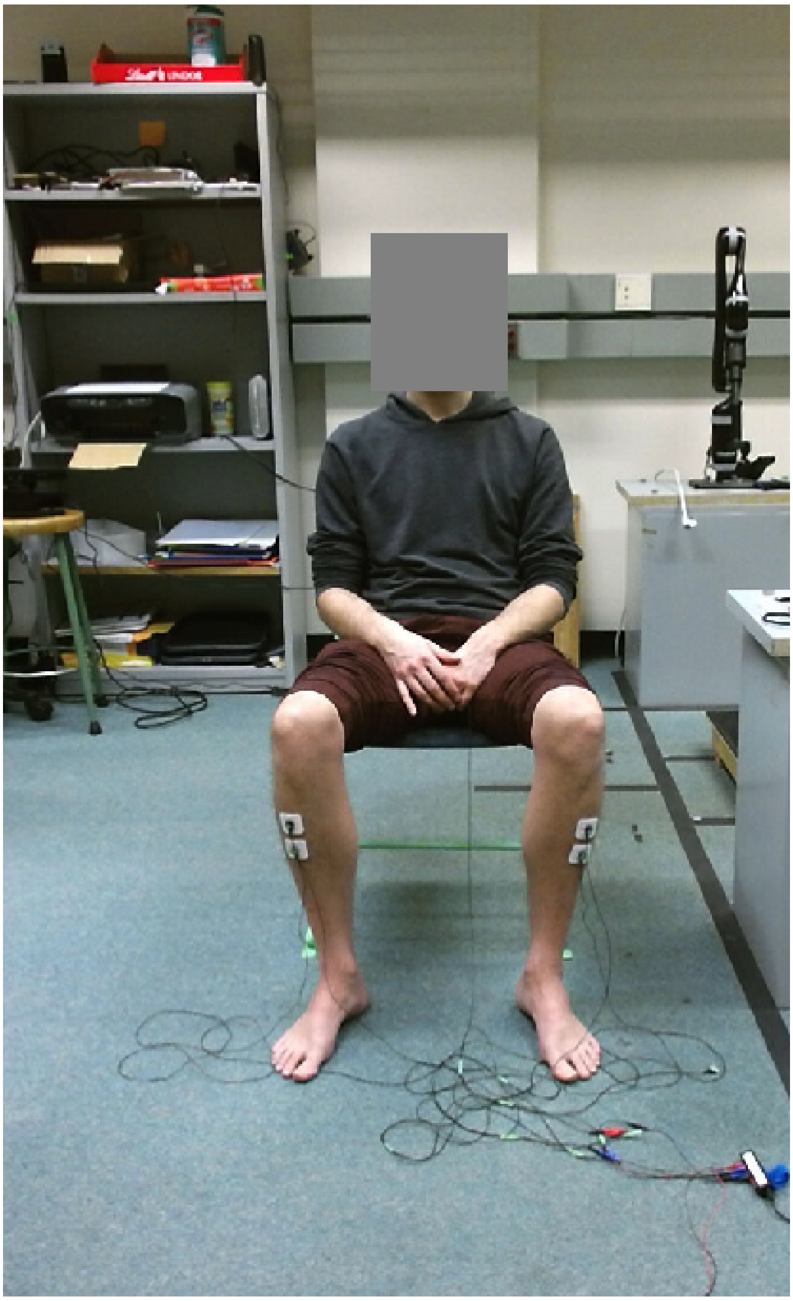

Describe everything you see in the snapshot.

____

Create a hierarchy: Rank your initial descriptions in the order you observed them. You may copy and paste your descriptions from the field above and add additional details as necessary.

1 ____________________________

2 ____________________________

3 ____________________________

4 __________________________________

5 __________________________________

6 __________________________________

7 __________________________________

8 __________________________________

9 __________________________________

10 __________________________________

11 __________________________________

12 __________________________________

13 __________________________________

14 __________________________________

15 __________________________________

16 __________________________________

17 __________________________________

18 __________________________________

19 __________________________________

20 __________________________________

21 __________________________________

22 __________________________________

23 __________________________________

24 __________________________________

25 __________________________________

26 __________________________________

27 __________________________________

28 __________________________________

29 __________________________________

30 __________________________________

List any additional descriptions in the space provided.

____
(Use numerals to separate descriptions (31., 32., ...))

This image is a snapshot in time. Describe what you would expect to happen next.

____
