# Supplementary material for: Fidgety Philip and the Suggested Clinical Immobilization Test: Annotation data for developing a machine learning algorithm
Source: Data Brief. 2021 Jan 17;35:106770. doi: 10.1016/j.dib.2021.106770 (PMC7851356; doi:10.1016/j.dib.2021.106770)
Supplement: Supplementary file 4 [file mmc4.pdf]

HMovements

a machine learning algorithm for detecting phenotypical sitting movement patterns in videos

User Guide

about

Purpose: the *HMovements* software is intended to assist clinicians with analyzing the body movement of patients with ADHD, restless legs syndrome/Willis Ekbom disease, and agitation syndrome (akathisia) during standardized sitting videos.

How it works: The user uploads a video of a patient to the software. Then, the software uses *OpenPose* — an open-source real time system for multi-person 2D pose detection — to process the video and digitally generate a skeletal overlay of the body, including major joints. The motion data is then exported into diagrams which visualize the frequency of movements observed in different regions of the body, based on pre-set thresholds. The user is also able to de-identify videos using a separate application within this software.

- The *HMovements* application can be downloaded here.
- The *HMovements* source code — for software developers to improve the algorithm — can be accessed on github.

Evaluation: The automated system has been evaluated against user-defined motion annotations. More details can be found in our two publications:

Beyzaei N, Bao S, Bu Y, Hung L, Hussaina H, Maher KS, Chan M, Garn H, Kloesch G, Kohn B, Kuzeljevic B, McWilliams S, Spruyt K, Tse E, Van der Loos HFM, Kuo C, Ipsiroglu OS. Is Fidgety Philip's ground truth also ours? The creation and application of a machine learning algorithm. Journal of Psychiatric Research. 2020 Dec;131:144-151. DOI: 10.1016/j.jpsychires.2020.08.033.[1,2]

Chan M, Tse E, Bao S, Berger M, Beyzaei N, Campbell M, Garn H, Hussaina H, Kloesch G, Kohn B, Kuzeljevic B, Lee Y-J, Maher KS, Carson N, Jeyaratnam J, McWilliams S, Karen Spruyt, Hendrik F. Machiel Van der Loos, Calvin Kuo, Osman S. Ipsiroglu. Fidgety Philip and the Suggested Clinical Immobilization Test: Annotation Data for Developing a Machine Learning Algorithm. Data in Brief. 2020 Dec.[2]

Correspinding authors:

[1]Calvin Kuo, School of Kinesiology, Faculty of Education and Department of Computer Science, Faculty of Science, University of British Columbia, Vancouver, BC, Canada. Email: calvin.kuo@ubc.ca

[2]Osman S. Ipsiroglu, H-Behaviours Research Lab, BC Children's Hospital Research Institute, Vancouver, BC, Canada; Department of Pediatrics, Faculty of Medicine, University of British Columbia, Vancouver, BC, Canada. Email: oipsiroglu@bcchr.ca

recommended specifications

Hardware for CPU OpenPose (slow)

**OS**	Windows 10
**Processor**	Intel® Core™ i5-4570 @ 3.20 GHz
**GPU**	Nvidia GeForce GTX 760
**Memory**	8GB
**Storage**	3GB

Hardware for GPU OpenPose (fast)

**GPU:**	OpenPose recommends any GPU with at least 10GB VRAM.

Note: This application cannot be used on a Mac.

Video Upload Requirements

- The video must only have **one** person in it
- The video must be in MP4 format and be ~30 frames per second.
- The video must be at least five seconds long.

set-up application

before you upload a patient video into the application, there is some prep work to be done.

1. Download the **HMovements** software folder to your computer, from here.
2. Locate the folder. This may be in the *Downloads* folder on your computer as a zipped folder. Once you find it, right click the *HMovements* folder and select 'extract here' to unzip the folder.
3. You should now see an unzipped version of the *HMovements* folder. — Click to open it. There should be an assortment of files inside. Do not alter the files within the HMovements folder
4. Click the *HMovements* application to open it
5. Now that the application is open, some file management prep-work needs to be done before uploading your patient video:
    - Consider where you want to store your analyzed video data. We would recommend storing this information on a secure drive.
    - Create a new folder in this location and label the folder so that you can find it later (e.g. 'HMovements_Data Export_Patient XX_Date').

⁎ When you upload your patient video, the software will ask you to select an empty folder to save your results to. This is the folder you should select.

⁎ Here is a list of files that will be created inside this folder.

- json folder contains the raw data outputted by OpenPose*
- config.json contains the configuration of your settings*
- data.json contains the data processed from the raw data by OpenPose*
- An AVI video file named skeleton.avi, the skeletal overlay video in AVI format
- A MP4 video file named skeleton.mp4, the skeletal overlay video in MP4 format*
- Analysis.npz
- SegmentEpochs.csv contains the epoch data for each body segments in CSV format
- SegmentMovements.csv contains the motion data for each body segments in CSV format
- SegmentEpochs.xiact

*Please do not remove or alter these files. If removed or altered, the application may not load the save folder properly.

Note: There are a few more items that could be added to this folder if the deidentification feature is used. The deidentification feature will be covered later.

upload video

once you have set-up your files, you are ready to upload your patient video. — this step might take a while.

1. Open the *HMovements* application. (Review set-up instructions on page 4).
2. Review the **two video upload options** (See *Figure 1*):
   - **Upload New Video to Process.** This option is intended for new video content.
   - **Upload Existing File.** This option is intended for videos you have previously uploaded to the application.

   Make sure to follow the **Video Upload Requirements** outlined on page 3.
3. Select an upload option.

   If you're **Uploading a New Video to Process:**
   - Select the folder you wish to save the data to. (Review set-up instructions on page 4)
   - Press continue and let the application process. While the video is processing, your computer will divert most of its computing power to the application. It is recommended to run the software overnight. There will be a loading screen indicating the percentage of completion.

   Note: It will take a long time to process depending on the specification of the computer. Computers without a dedicated GPU that has at least 8GB of memory will take hours just to process a one-minute video. The reason is because OpenPose requires an exorbitant amount of processing power to run.

   If you're **Uploading an Existing File:**
   - Select the folder where you saved the video previously and the application will load it into the main screen.

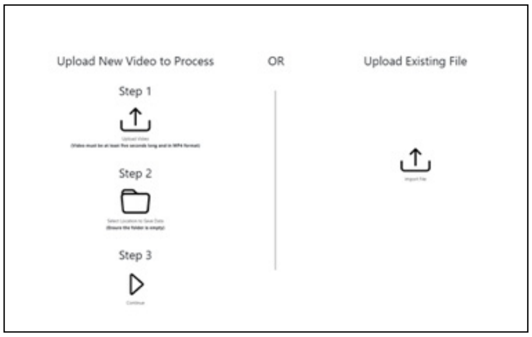

Figure 1: Start Screen

view results main interface

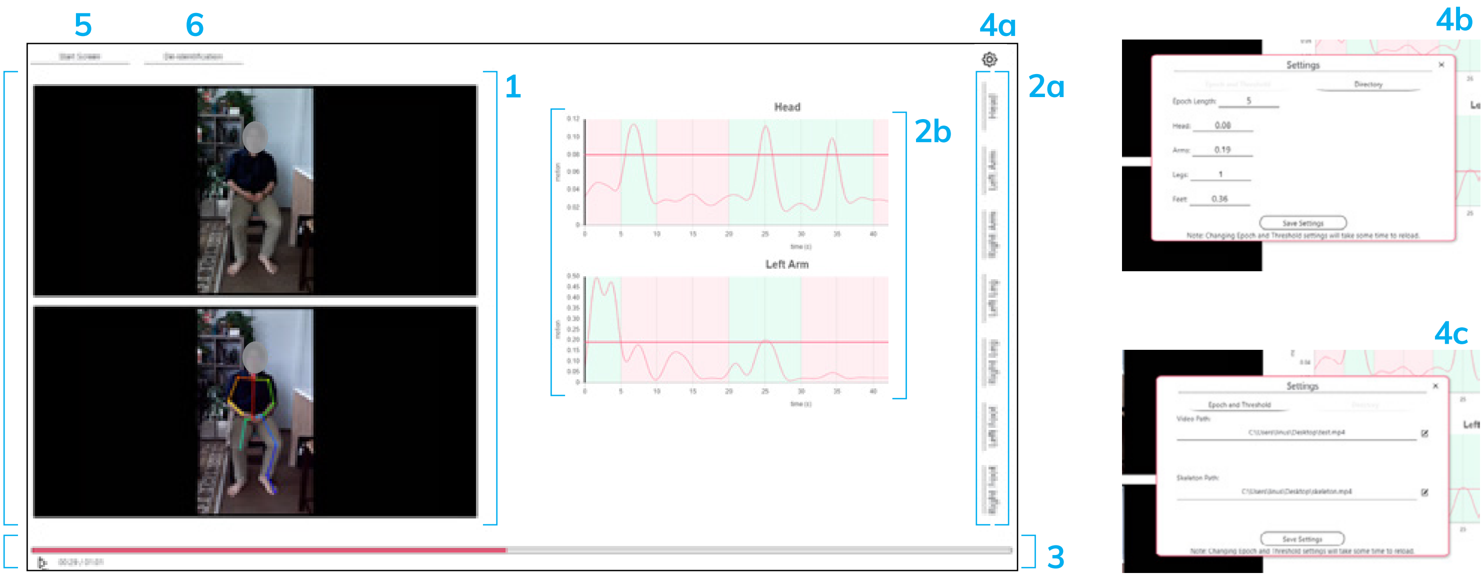

Figure 2: Sample results screen with a team member (CK).

This is the main user interface (See Figure 2) that will display after the video has been processed or if an existing file has been loaded. It consists of three areas:

1. **Video Display.** The top video displays the raw video that the user inputted, and the bottom video displays the rendered skeleton video.
2. **Body Part Diagrams:**

   a. The right side of the screen consists of tabs. To reveal a specific diagram, select the name of a body part you want to view. To close this view, de-select the name. You can view multiple diagrams at once.

   b. This view shows the selected diagrams.
3. **Video Controls.** The bottom of the screen is the video control that governs the two videos. The user can click and drag the video scroll bar to view different times of the video.
4. **Settings:**

   a. On the top right of the screen, there is a gear icon. When the gear icon is clicked, the settings menu would pop up in the middle of the screen.

   b. Epoch and Threshold tab is the tab where you can change epoch and threshold settings. Be aware that changing settings in this tab would take some time to reload as the application would have to process the raw json and regenerate a new data.json file.

   c. Directory tab is the tab where you can change the paths for raw video and the skeleton video path. Changes made in this tab are instantaneous. Do not move the videos from the directory manually in the settings or else the user interface will not be able to launch.
5. **Start Screen:** By pressing this, you can return to the main menu and re-upload or reprocess a video
6. **De-identification.** See page 8 for more details

view results diagram view

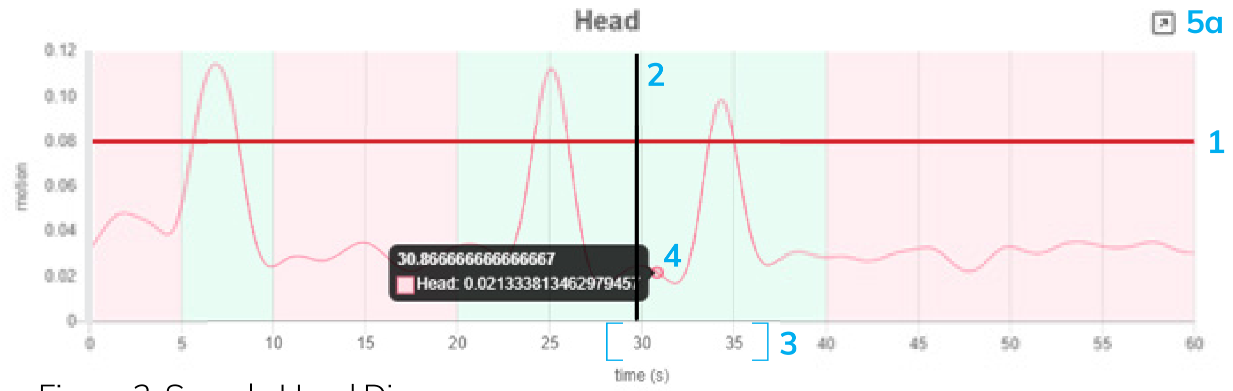

Figure 3: Sample Head Diagram.

The diagrams visualize the motion data extracted from the inputted video:

1. **Threshold.** This line stretching across the diagram indicates the threshold for that body part. The threshold can be changed in settings.
2. **Time Marker.** This indicates the current video time.
3. **Epoch length.** The default epoch length is five seconds, which is also the step size of the x-axis. Within each epoch, the colour of the diagram would be green if the motion exceeds the threshold and red otherwise.
4. **Data Values.** The user can hover over data points on the diagram to see the value in depth. This function can only be used when the video is paused as the diagram is constantly refreshing.
5. **Reconfigure Display:**

   a. On the top right of each diagram, there is a small arrow button. When you hover over this button, the words *Pop Out* will appear

   b. When selected, the diagram will pop out in a new window. This will allow you to re-size the diagram, to optimize viewing.

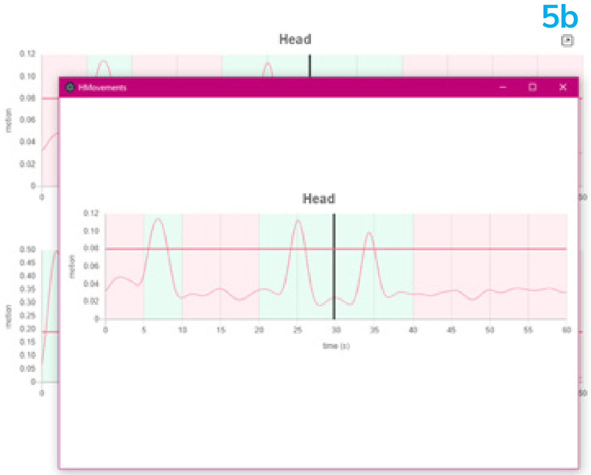

Figure 4: Pop-out view

de-identify video

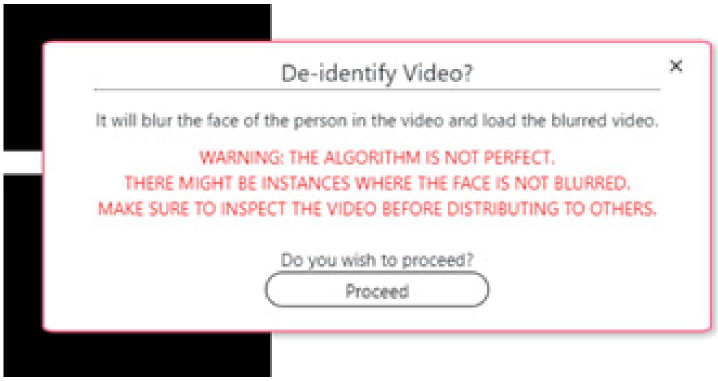

Figure 5: De-identification feature.

The de-identification process takes the video uploaded by the user and blurs it using an algorithm built around OpenCV.

This option can be found at the top left of the screen labeled 'De-identification'. When the button is pressed, a dialog box will pop-up detailing the de-identification process as well as displaying an option to proceed with the de-identification.

After the de-identification completes, the newly blurred video will be loaded into the video player. (The skeleton version would also be blurred as well).

The user can find the blurred videos in the saved folder. The video and skeleton path in the settings will also be changed to the paths of the blurred videos. If you want to load the original video, you can change the video path to the original path.

Note: It will take some time (around eleven minutes for a one-minute video, but this will vary depending on the specifications of your hardware) for the de-identification process to complete depending on the length of the video.

Warning: The algorithm is not perfect. There might be frames where the faces are not blurred. We recommend users view the processed video to ensure full de-identification before dissemination.

If there are gaps in blurring, use other software (e.g., Adobe Premiere, Camtasia) to manually add blurring.

FAQs

Why is there a discrepancy between diagram length and video length?

The diagram length is the closest multiple of the epoch length to the video length.

e.g. if the video length is one minute and three seconds and the epoch length is five seconds, then diagram length would be one minute.

If I have any questions or feedback about the algorithm, who should I contact?

Please contact Dr. Calvin Kuo at calvin.kuo@ubc.ca.
